# Simulated maternal stress reduces offspring aerobic swimming performance in Pacific salmon

**DOI:** 10.1093/conphys/coz095

**Published:** 2019-12-18

**Authors:** Amanda I Banet, Stephen J Healy, Erika J Eliason, Edward A Roualdes, David A Patterson, Scott G Hinch

**Affiliations:** 1 Department of Biological Sciences, California State University, Chico, 400 W. First Street, Chico, CA 95929, USA; 2 Pacific Salmon Ecology and Conservation Laboratory, Department of Forest and Conservation Sciences, University of British Columbia, 2424 Main Mall, Vancouver, BC, V6T 1Z4, Canada; 3 Department of Ecology, Evolution, and Marine Biology, University of California, Santa Barbara, Santa Barbara, CA 93106, USA; 4 Department of Mathematics and Statistics, California State University, Chico, 400 W. First Street, Chico, CA 95929, USA; 5 Fisheries and Oceans Canada, Science Branch, Pacific Region, Co-operative Resource Management Institute, School of Resource Environmental Management, Simon Fraser University, 8888 University Drive, Burnaby, BC, V5A 1S6, Canada

**Keywords:** cortisol, maternal effects, *Oncorhynchus*, Pacific salmon, respirometry, swimming performance

## Abstract

Pacific salmon routinely encounter stressors during their upriver spawning migration, which have the potential to influence offspring through hormonally-mediated maternal effects. To disentangle genetic vs. hormonal effects on offspring swimming performance, we collected gametes from three species of Pacific salmon (Chinook, pink and sockeye) at the end of migration and exposed a subset of eggs from each female to cortisol baths to simulate high levels of maternal stress. Fertilised eggs were reared to fry and put through a series of aerobic swim trials. Results show that exposure to cortisol early in development reduces maximum oxygen consumption while swimming, and decreases aerobic scope in all three species. Resting oxygen consumption did not differ between cortisol and control treatment groups. We also examined several metrics that could influence aerobic performance, and found no differences between treatment groups in haematocrit%, haemoglobin concentration, heart mass, citrate synthase activity or lactate dehydrogenase activity. Though it was not the focus of this study, an interesting discovery was that pink salmon had a higher MO_2max_ and aerobic scope relative to the other species, which was supported by a greater haematocrit, haemoglobin, a larger heart and higher CS activity. Some management and conservation practices for Pacific salmon focus efforts primarily on facilitating adult spawning. However, if deleterious effects of maternal stress acquired prior to spawning persist into the next generation, consideration will need to be given to sub-lethal effects that could be imparted onto offspring from maternal stress.

## Teaser

Lay summaries We investigated whether maternal stress in Pacific salmon can influence aerobic swimming performance of offspring, through stress hormones deposited into the eggs. We found that offspring exposed to higher levels of the stress hormone cortisol at fertilisation had poorer aerobic swimming performance, which may reduce survival in the wild.

## Introduction

An organism’s phenotype is influenced not only by genetics and environmental factors, but also by maternal effects, whereby the mother’s genotype, phenotype and body condition indirectly affect offspring in utero (Wolf and Wade, 2009). Historically, maternal effects were thought to have only a minor role in ecology, but in recent years their importance has become increasingly clear (Bernardo, 1996; Ho and Burggren, 2010). In many cases, maternal effects are considered adaptive (Mousseau and Fox, 1998). For example, female guppies (*Poecilia reticulata*) on a restricted diet produce larger offspring (Reznick *et al.*, 1996), which are better adapted to survive in conditions with low food availability (Hutchings, 1991). In another study, *Daphnia cucullata* offspring whose mothers had been exposed to a predator had longer helmets (an inducible defence against predators) than offspring whose mothers were not exposed to predators (Agrawal *et al.*, 1999).

The aforementioned studies show that maternal effects, or more generally, parental effects, can provide a mechanism for an organism to be prepared for variable environmental conditions. However, if a population has not experienced similar variation in environmental conditions over evolutionary time, it is unlikely that an adaptive response will have had time to evolve. In such cases, maternal effects may instead be a non-adaptive physiological consequence of environmental conditions (Marshall and Uller, 2007). This is of particular relevance because anthropogenic habitat degradation and climate change are increasingly exposing animals to rapidly changing environmental conditions that fall outside of their historic range. Thus, understanding maternal effects on offspring characteristics is a critical step to understanding how a given population may respond to these changes—an important consideration for conservation and management decisions (Charmantier and Garant, 2005; Burt *et al.*, 2010).

Pacific salmon encounter numerous stressors during their upriver spawning migration, including warm water temperatures and high encountered flows. The direct effects of these stressors have been widely documented and in many instances are becoming more severe as climates continue to warm (Martins *et al.*, 2012a). Adult pink salmon (*Oncorhynchus gorbuscha*) and sockeye salmon (*Oncorhynchus nerka*) that experienced high temperatures during a laboratory-simulated migration had an increase in physiological biomarkers of stress compared to their low-temperature counterparts, including changes in protein folding, protein synthesis, metabolism, oxidative stress and ion transport (Jeffries *et al.*, 2014). Simulated fisheries capture and handling produced a major stress response in these same species, increasing activation of genes involved in cellular stress, and plasma levels of stress indicators such as cortisol and lactate (Donaldson *et al.*, 2014). Cook et al. (2014) found that after being captured, adult sockeye salmon with higher levels of physiological stress exhibited reduced migration success. A number of other studies have found additional detrimental patterns of adult salmon stress (Bradford *et al.*, 2010; Veldhoen *et al.*, 2010; Martins *et al.*, 2012b; Eliason *et al.*, 2013; Gale *et al.*, 2014; Raby *et al.*, 2015).

Environmental stressors also have the potential to indirectly affect the next generation via hormonally-mediated maternal effects. Maternal cortisol, a glucocorticoid hormone involved in the stress response, can be transferred from mother to egg (Stratholt *et al.*, 1997) and may affect offspring characteristics later in life. For example, Tierney *et al.* (2009) examined the influence of maternal condition on offspring locomotor performance in sockeye salmon. They examined swimming performance differences between offspring reared from eggs that were collected from females that were classified as spawn-ready (good condition) or moribund (poor condition) at the end of their spawning migration. Moribund females had higher plasma cortisol and blood lactate levels than spawn-ready females, indicating higher levels of stress. The study found that offspring from moribund females had poorer performance on a number of swimming metrics, including reduced endurance (a measure of aerobic performance), poorer schooling performance and a shorter distance travelled when startled. These differences may be due to exposure to maternal cortisol early in development, but the design of the study allows for alternative explanations. For example, it is possible that the females had genetic differences in swimming ability that made some of them less equipped to deal with the demands of migration, and these genetic differences in swimming ability were passed on to their offspring.

The aim of this study is to disentangle the effect of cortisol exposure early in development on offspring aerobic swimming performance from other aspects of maternal identity. We first compare aerobic swimming performance between siblings that either have or have not been exposed to simulated maternal stress via a cortisol bath at fertilisation. If exposure to maternal cortisol contributed to the results found by Tierney *et al.* (2009), then fishes that were exposed to the cortisol baths should exhibit a reduction in aerobic swimming performance as compared to control fish. We then examine the mechanistic underpinnings of the differences in swimming performance by comparing physiological traits that determine an organism’s ability to use oxygen to produce energy.

## Materials and methods

### Study system

**Figure 1:**
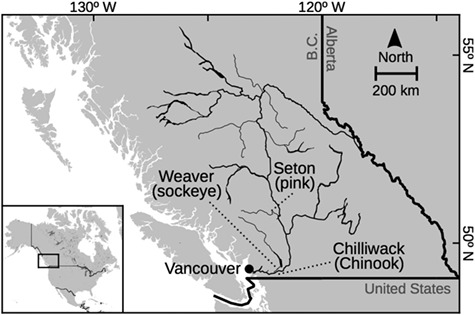
Map of the Fraser River Drainage, British Columbia, Canada. Spawning locations of the three populations of Pacific salmon used in this study are labeled. Respirometry trials were conducted at the University of British Columbia campus in Vancouver, B.C., Canada.

This study examines three species of anadromous, semelparous Pacific salmon from the Fraser River Drainage: sockeye salmon (*O. nerka*) from Weaver Creek, Chinook salmon (*Oncorhynchus tschawyscha*) from the Chilliwack River Hatchery and pink salmon (*O. gorbuscha*) from the Seton River (Fig. 1). These species have ecological, economic and cultural value in the North Pacific, making them the focus of conservation and sustainable management efforts throughout their range (Quinn, 2005).

### Gamete collection and rearing

This study followed animal use protocols approved by the Canadian Council on Animal Care and the University of British Columbia Animal Care Committee. Adult fishes were sacrificed by manually applied percussive stunning followed by pithing. Gametes were then manually stripped from the fish into sterile plastic containers by palpating the abdomen. Unfertilised gametes from each individual were housed in separate, labelled container(s) and placed in an insulated cooler for transport. Eggs were housed no more than two layers thick in the containers and milt was housed in containers that allowed a large surface area, in order to ensure oxygen exchange. The transport cooler contained several commercial ice packs, which were separated from the gamete containers with a layer of polystyrene foam. After collection, gametes were transported to the University of British Columbia aquatic facility for fertilisation. Gametes were collected in autumn 2013 for all species. Collection occurred on 9 October for Seton River pink salmon, 21 October for Chilliwack River hatchery Chinook salmon and 23 October for Weaver Creek sockeye salmon.

Fertilisations occurred within 24 hours of gamete collection and followed methods similar to Sopinka *et al.* (2015). Fourteen grams of eggs from a single female were weighed and measured into a glass jar and fertilised with 0.15 ml of milt from a single male. This was done in duplicate to create matched control and cortisol-treated full sibling crosses. Initially, 30 ml of water was added to each jar to activate the sperm. After 2 minutes, an additional 370 ml of water was added to each jar. Cortisol treatment fertilisations were conducted using dechlorinated facility water that had been dosed to contain 1000 ng/ml of cortisol (H4001; Sigma). Ninety-five percent ethanol was needed to dissolve the cortisol, which resulted in a 0.002% final concentration in the treated water. Control treatment fertilisations were conducted using dechlorinated facility water that contained 0.002% ethanol, in order to control for any effects of ethanol exposure. The concentration of cortisol in the treated water was chosen to match previous studies that used cortisol baths to increase egg cortisol within an ecologically relevant range (Auperin and Geslin, 2008; Sopinka *et al.*, 2015). This process resulted in nine unique crosses for Chinook salmon, nine for pink salmon and 12 for sockeye salmon.

After 2 hours, fertilised eggs from each jar were rinsed and transferred to a flow through basket and incubated at 9.5 ± 1°C in temperature-controlled heath stacks until emergence. For sockeye and pink salmon, a subset of eggs were rinsed and collected two hours after fertilisation in order to confirm that treatment egg cortisol levels increased to a level within an ecologically relevant range. Cortisol assays did indeed confirm this (see online supporting information for detail). Dead eggs were recorded and removed from heath stacks daily. Fertilisation-to-hatch duration was approximately 57 days (541 accumulated thermal units, or ATU) for pink salmon, 54 days (513 ATU) for Chinook salmon and 65 days (617.5 ATU) for sockeye salmon. After button-up, emergent fish were transferred to 1000-l flow-through troughs, which had mesh dividers to separate families and treatment groups. Fish were fed fishmeal *ad libitum* until ~48 hours prior to aerobic trials. Light cycle throughout the experiment mimicked a natural photoperiod (49.2606°N, 123.2460°W). Water temperature of the flow-through troughs that housed the fish varied depending on the environmental temperature, and ranged from 4.5 to 11.0°C over the course of the experiment.

### Aerobic swimming trials

Aerobic trials were conducted from 7 April 2014 to 25 June 2014 (fish mass: Chinook salmon, mean and 95% CI = 0.7161 ± 0.081 g, range = 0.4072–1.0376 g; pink salmon, mean and 95% CI = 0.4291 ± 0.082 g, range = 0.2517–0.6286 g; sockeye salmon, mean and 95% CI = 0.7421 ± 0.065 g, range = 0.5191–1.0148 g). Aerobic performance was measured using two Loligo Systems 170 ml mini-swim respirometers (Loligo Systems, Viborg, Denmark) following methods similar to Svendsen *et al.* (2013) with minor modifications. Two respirometers allowed two trials to be run per day, including one control fish and one cortisol treatment fish from a full-sibling pair. The respirometers were identical models, but as an extra precaution, we alternated treatment groups between the respirometers to prevent any differences in the equipment from confounding our data. For each trial, a fish was introduced to the working section of the swimming respirometer and allowed to acclimate overnight for a minimum of 15 hours in water moving at 0.5 cm/second. This speed allowed water to mix inside the tunnel, but the fish was able to rest on the bottom of the respirometer without actively swimming. Fully aerated water was flushed through the respirometer during the acclimation period. Water temperature was maintained at 7 ± 0.1°C using aquarium chillers and heaters regulated by a thermostat. In order to reduce disturbances, we covered the respirometer with a layer of black plastic with a small opening that allowed observation. After the acclimation period, the respirometer chamber was sealed and we measured resting oxygen consumption (MO_2rest_). MO_2rest_ has been defined various ways in the literature (Munday *et al.*, 2012; Schaeffer *et al.*, 2012; Eliason *et al.*, 2013); for the purposes of this study, we define MO_2rest_ as the mean oxygen consumption of a fish resting on the bottom of the tunnel over a 30-minute period after at least 15 hours of acclimation. After MO_2rest_ was measured, we exposed the fish to incremental increases of water velocity of 2 cm·second^−1^ every 2 minutes. When the fish appeared to be approaching its maximum swimming capacity (e.g. it exhibited early stages of bursting and gliding), the respirometer chamber was sealed for measurement, and we reduced the frequency of water velocity increases to every 5 minutes. The trial continued until the fish fatigued and fell against the back of the tunnel for a minimum of 10 seconds. Maximum oxygen consumption (MO_2max_) was estimated using the mass-specific rate of oxygen consumption obtained for the final 5-minute period of the trial prior to fatigue, with the exception of four fish that fatigued before 5 minutes of data could be collected. This included one control chinook (3m37s), one cortisol-treated chinook (3m20s), one control sockeye (3m14s) and one cortisol-treated sockeye (3m0s). For these fish, MO_2max_ was estimated using the truncated data available before fatigue. For all trials, including those from fish that fatigued before 5 minutes of MO_2max_ data could be collected, the *R*^2^ value of oxygen consumption over time was greater than 0.98. This strong linear relationship suggests that the data collected during the four truncated trials were commensurate to those in which we were able to collect a full 5 minutes of data. The slope of oxygen consumption over time was used in conjunction with fish mass and swim tunnel volume to calculate mass-specific oxygen consumption. Total sample sizes are available in Table 1. Aerobic scope was calculated as the difference between the MO_2max_ and MO_2rest_. Trials were excluded from the study if the fish defecated in the tunnel during acclimation, or if the fish was actively moving during the resting measurement. Background oxygen loss was checked routinely throughout the study using empty respirometers for 30 minutes and determined to be negligible.

**Table 1:** Results from linear mixed model analyses. Dependent variables are listed in the left column and fixed effects are listed across the top of the table. Bold text indicates statistical significance. A dash indicates a variable that was not included in the model. Sample sizes are listed in the following format: chinook control, chinook cortisol: pink control, pink cortisol: sockeye control, sockeye cortisol

	Treatment	Species	Fish mass	Block (Respirometer)	Species × treatment
MO_**2rest**_	*F*_1, 26_ = 0.03	*****F*****_****2, 27****_ ****= 9.67****	-	*F*_1, 26_ = 0.02	*F*_2, 26_ = 0.11
*n* = 9,9:9,9:12,12	*P* = 0.86	*P* ****= 0.0007****	-	*P* = 0.88	*P* = 0.90
**MO**_**2max**_	*****F*****_****1, 26****_ ****= 30.67****	*****F*****_****2, 27****_ ****= 47.59****	****-****	*****F*****_****1,26****_ ****= 8.03****	*F*_2, 26_ = 2.69
*n* = 9,9:9,9:12,12	*P* ****< 0.0001****	*P* ****< 0.0001****	****-****	*P* ****= 0.009****	*P* = 0.09
**Aerobic scope**	*****F*****_****1, 26****_ ****= 29.58****	*****F*****_****2, 27****_ ****= 32.44****	****-****	*****F*****_****1.26****_ ****= 7.02****	*F*_2, 26_ = 2.40
*n* = 9,9:9,9:12,12	*P* ****< 0.0001****	*P* ****< 0.0001****	****-****	*P* ****= 0.01****	*P* = 0.11
**Haematocrit**	*F*_1, 64.46_ = 0.37	*****F*****_****2, 28.26****_ ****= 33.50****	*F*_1, 89.23_ = 3.70	-	*F*_2, 67.22_ = 0.71
*n* = 17,18:19,17:14,14	*P* = 0.54	*P* ****< 0.0001****	*P* = 0.06	-	*P* = 0.49
**Haemoglobin**	*F*_1, 65.45_ = 2.47	*****F*****_****2, 28.18****_ ****= 54.37****	*****F*****_****1, 83.32****_ ****= 9.25****	-	*F*_2, 68.98_ = 0.98
*n* = 18,18:18,17:14,14	*P* = 0.12	*P* ****< 0.0001****	*P* ****= 0.003****	-	*P* = 0.38
**Heart mass**	*F*_1, 65.72_ = 0.05	*****F*****_****2, 28.27****_ ****= 40.88****	*****F*****_****1, 80.90****_ ****= 78.84****	****-****	*F*_2, 68.70_ = 0.46
*n* = 18,18:18,17:14,14	*P* = 0.83	*P* ****< 0.0001****	*P* ****< 0.0001****	-	*P* = 0.63
**CS activity**	*F*_1, 103.16_ = 0.65	*****F*****_****2, 19.06****_ ****= 25.37****	-	-	*****F*****_****2, 103.16****_ ****= 3.93****
*n* = 16,17:24,24:23,24	*P* = 0.42	*P* ****< 0.0001****	-	-	*P* ****= 0.02****
**LDH activity**	*F*_1, 107_ = 0.00	*****F*****_****2, 19****_ ****= 40.06****	-	-	*****F*****_****2, 107****_ ****= 4.49****
*n* = 18,18:24,24:24,24	*P* > 0.99	*P* ****< 0.0001****	-	-	*P* ****= 0.01****

### Blood and heart mass

Haematocrit and haemoglobin data were collected from a second subset of experimental fish, which were not used in the aerobic trials. Fish were euthanized in a lethal dose of MS-222 (250 mg L^−1^ buffered to pH 7.0; Sigma Aldrich, Darmstadt, Germany), dried with a Kimwipe, and weighed to the nearest ten-thousandth of a gram. The tail was then severed posterior to the anal fin in order to access blood from the caudal vein. Blood for haemoglobin analysis was collected on cuvette, measured using a HemoCue™ Hb 201+ Analyzer (Hemocue AB, Ängelholm, Sweden), and calibrated following methods in Clark *et al.* (2008). Blood for haematocrit analysis was collected in a heparinized microhaematocrit tube, spun in a microhaematocrit centrifuge at 8000 rpm for 5 minutes, and packed-cell volume was measured using a microhaematocrit reader card.

Heart mass was collected from a third subset of experimental fish. Fish were euthanized in MS-222, dried with a Kimwipe and weighed to the nearest ten-thousandth of a gram. The heart was then carefully removed via dissection under a stereomicroscope at 10× magnification, dabbed gently on a Kimwipe to remove excess fluids, and weighed to the nearest ten-thousandth of a gram.

### Enzyme assays

Enzyme assays were conducted on tail muscle samples from a fourth subset of experimental fish. Fish were euthanized in MS-222, frozen in liquid nitrogen, and transferred to a −80°C freezer for later analysis. These samples were collected during the same time frame as the aerobic trials. Activity of citrate synthase (CS), an enzyme involved in the citric acid cycle and lactate dehydrogenase (LDH), an enzyme that catalyses the interconversion of pyruvate and lactate, were measured following methods similar to Dalziel and Schulte (2012), including pilot assays to determine appropriate substrate concentrations for analysis. Briefly, muscle tissue posterior to the dorsal fin was dissected from the fish, and the tail and skin were removed from the sample. The muscle was weighed and added to 10 volumes of buffer (50 mmol L^−1^ Hepes, 1 mmol L^−1^ EDTA and 0.1% Triton X-100; pH 7.4) with 0.5 mm zirconium oxide beads equal to 3× the mass of the muscle tissue. This sample was homogenized at 1700 rpm (Geno/Grinder 2010, SPEX SamplePrep, Stanmore, UK) for 2.5 minutes, put on ice for 2.5 minutes, and re-homogenized for another 2.5 minutes. An additional 1:1 volume of homogenization buffer was then added to the sample, and it was refrozen at −80°C. Enzyme activity was determined at 25°C using a temperature-controlled plate spectrophotometer. For CS assays, we took 25 μL of frozen homogenate, thawed it on ice, and added 25 μL of Tris buffer before pipetting 10 μL of the diluted sample into plate wells. The CS assay was run with final concentrations of 0.15 mmol L^−1^ DTNB, 0.15 mmol L^−1^ Acetyl CoA, and 0.5 mmol L^−1^ oxaloacetate in 50 mmol L^−1^ Tris. For LDH assays, we took 4 μL of frozen homogenate, thawed it on ice and added 46 μL of Tris buffer before pipetting 10 μL of the diluted sample into plate wells. The LDH assay was run with final concentrations of 5 mmol L^−1^ NADH and 25 mmol L^−1^ pyruvate in 50 mmol L^−1^ Tris. All assays were measured in triplicate, and background reaction rates of buffer with no substrate present were subtracted from measured values.

### Statistical analyses

All statistical analyses were conducted using R (R Core Team, 2016). We used linear mixed models to test for differences between treatment groups for each of our target variables (MO_2rest_, MO_2max_, aerobic scope, heart mass, haematocrit, haemoglobin, CS and LDH). Sibling pairs were treated as random variables to account for correlations between observations due to relatedness. *P*-values for mixed models were estimated via approximate *F*-tests with Kenward–Roger approximated degrees of freedom (Kenward and Roger, 1997). Species, treatment and the interaction between species and treatment were included as fixed effects in analyses for MO_2rest_, MO_2max_ and aerobic scope. Respirometer was also included as a blocking variable in these analyses. We did not include fish mass in the aerobic analyses reported below; however, we did run an additional analysis (not reported) that included fish mass as a factor and found that there were no qualitative differences in terms of significance for any of the other covariates included in the mixed models. Species, fish mass, treatment and the interaction between species and treatment were included as fixed effects in analyses for heart size, haematocrit and haemoglobin. Species, treatment and the interaction between species and treatment were included as fixed effects in analyses for CS and LDH. Alpha was set at 0.05 for all analyses. Sample sizes are available in Table 1. Though it was not the main focus of the study, we examined differences between species by conducting hypothesis tests of estimated marginal means while controlling for multiple comparisons using Tukey’s method for *P*-value adjustments (Searle *et al.*, 1980; Lenth, 2018).

## Results

### Aerobic performance

**Figure 2:**
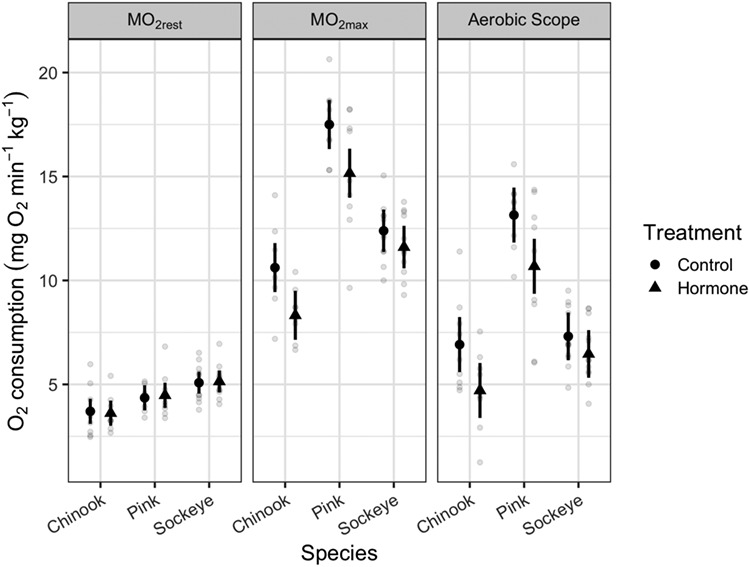
Aerobic performance of three species of Pacific salmon. Estimated marginal means averaged over block (respirometer) and 95% confidence intervals are shown in black. Grey circles represent individual fish. Cortisol exposure had no effect on resting oxygen consumption (MO_2rest)_), but significantly reduced maximum aerobic performance (MO_2max_) and aerobic scope.

MO_2rest_ was significantly influenced by species, but it was not significantly affected by cortisol treatment, block (respirometer), or the interaction between species and treatment (Table 1, Fig. 2). Tukey *post hoc* tests of the estimated marginal means show that sockeye salmon had a higher MO_2rest_ than chinook salmon in both the control and cortisol fish (Online supplement Tables S1 and S2).

MO_2max_ and aerobic scope were both significantly lower in cortisol treated fish, and there were significant effects of species and block (respirometer) (Table 1, Fig. 2). All species showed a similar response to the cortisol treatment (Table 1, Fig. 2 and online supplement Table S1). Tukey *post hoc* tests of the estimated marginal means (Online supplement Tables S1 and S2) show that pink salmon had a higher MO_2max_ and aerobic scope than Chinook and sockeye salmon in both control and cortisol-treated groups. In cortisol-treated fish, sockeye salmon had a higher MO_2max_ than Chinook salmon.

### Blood and heart mass

**Figure 3:**
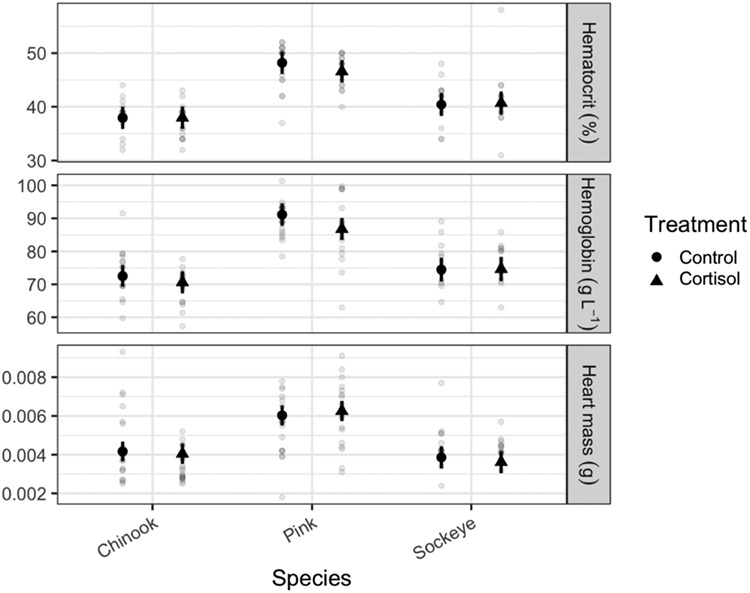
Blood and heart metrics of three species of Pacific salmon. Estimated marginal means and 95% confidence intervals are shown in black. Grey circles represent individual fish. Cortisol exposure did not significantly affect these metrics.

Haematocrit, haemoglobin and heart mass all showed similar patterns to one another (Table 1, Fig. 3 and online supplement Table S1). Species differed significantly for all three variables. Larger fish had significantly higher haemoglobin levels, and a larger heart mass. None of these metrics were affected by cortisol treatment or the interaction between species and treatment. Tukey *post hoc* tests of the estimated marginal means (Online supplement Tables S1 and S2) show that pink salmon had higher haematocrit and haemoglobin levels, as well as a larger heart mass relative to their body mass, as compared to their Chinook or sockeye counterparts in both control and cortisol treatment groups.

### Enzyme assays

**Figure 4:**
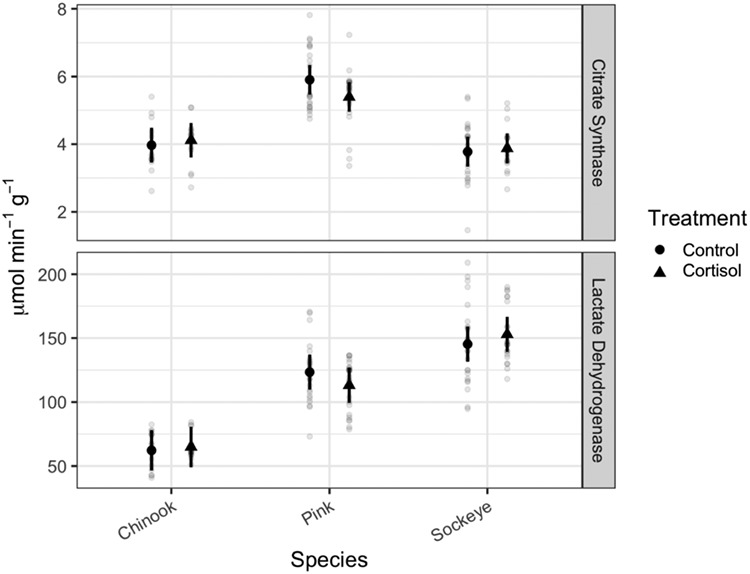
Enzyme activity per gram of tissue in the caudal muscle of three species of Pacific salmon. Estimated marginal means and 95% confidence intervals are shown in black. Grey circles represent individual fish. Cortisol exposure did not significantly affect these metrics.

There was no effect of cortisol treatment on CS or LDH activity; however, species and the interaction between treatment and species both had a significant influence (Table 1, Fig. 4 and online supplement Table S1). Tukey *post hoc* tests of the estimated marginal means (Online supplement Tables S1 and S2) show that pink salmon had higher CS activity than their Chinook or sockeye counterparts in both control and cortisol treatment groups. Control sockeye salmon and pink salmon both had higher LDH activity than control Chinook salmon. In the cortisol treatment group, sockeye salmon had higher LDH activity than pink salmon or Chinook salmon, and pink salmon had higher LDH activity than Chinook salmon.

## Discussion

This study shows that exposure to cortisol early in development can have persistent effects on juvenile Pacific salmon aerobic performance after hatch. Cortisol is released into the circulatory system of fish as part of the neuroendocrine response to stress (Barton, 2002), and previous work has found that increases in maternal plasma cortisol can translate to higher egg cortisol levels (Stratholt *et al.*, 1997). In all three species examined in our study, juvenile fish that were exposed to increased cortisol during embryonic development exhibited lower maximum oxygen consumption and aerobic scope than control fish, but maintained similar resting oxygen consumption. These results indicate that maternal stress during upriver migration could lead to impaired offspring performance across a range of species.

Contrary to expectations, we did not find differences between treatment groups in the oxygen-transport metrics that we measured. However, this does not preclude the possibility that cortisol may have an effect on these metrics under different circumstances, or on different oxygen transport metrics. For example, egg cortisol levels in our study were relatively high before treatment (see online supporting information); it is possible that cortisol does influence the oxygen delivery metrics we examined, but the effect was saturated at the levels examined in our study and could not be further enhanced. Additionally, our work focused on only a subset of traits that are involved in oxygen transport; there are a number of other variables that future work could examine. For example, cardiac performance, gill surface area and variation in other haemoglobin characteristics such as oxygen-binding affinity could also influence aerobic performance (Weibel, 1984; Weber, 2000). Similarly, other metrics such as mtDNA copy number and cardiolipin content have been used to estimate mitochondrial content in vertebrates, and there is no clear consensus on which metrics best represent actual mitochondrial performance (Kraffe *et al.*, 2007; Porter and Wall, 2012). Examination of these, and other factors involved in oxygen transport and use, will provide a more complete picture of how cortisol exposure during embryonic development affects Pacific salmon. Notably, we did find differences in MO_2max_ and aerobic scope across species, which were clearly associated with differences in oxygen transport metrics. Specifically, in both treatment groups, pink salmon had a higher MO_2max_ and aerobic scope relative to the other species, which was supported by greater haematocrit, haemoglobin, a larger heart and higher CS activity.

The differences we found in aerobic performance between treatment groups may also be mediated via the effects of egg cortisol on fish behaviour and motivation. Multiple studies have examined the relationship between prenatal stress and behaviour in fishes. In three-spined stickleback (*Gasterosteus aculeatus*), offspring from mothers that were exposed to high levels of a predation stressor exhibited less successful anti-predator behaviours (McGhee *et al.*, 2012), and were slower in learning tasks that paired certain cues with a food reward (Roche *et al.*, 2012). Atlantic salmon (*Salmo salar*) offspring from mothers that received an intraperitoneal cortisol implant prior to egg collection exhibited more unsuccessful feeding attempts, showed higher levels of aggression when they had established social dominance in a group, and spent less time moving when subjected to an acute confinement stressor (Eriksen *et al.*, 2011). In studies that simulated maternal stress via egg cortisol baths, coho salmon (*Oncorhynchus kisutch*) offspring showed increased social dominance and boldness when challenged with a conspecific intruder or predator, respectively (Sopinka *et al.*, 2015) and brown trout (*Salmo trutta*) were more aggressive and showed reduced learning in a maze test (Sloman, 2010). Though the goal in many respirometry studies is to get accurate representations of maximum physiological performance, behavioural differences in fish can influence oxygen consumption measurements (Clark *et al.*, 2013).

Cortisol may not be the only component of the stress response that can be transmitted between generations. In one recent study, sockeye salmon (*O. nerka*) adults were subjected to a chronic chase stressor for several weeks prior to spawning. Offspring from these fish swam for shorter durations and initiated burst swimming more often than fish from a control group, despite no detectable differences in egg cortisol levels (Sopinka *et al.*, 2014). The authors of the study hypothesized that other egg hormones associated with the maternal stress response, as well as heritable stress-induced epigenetic effects, may have contributed to their results. As such, we caution that while this study specifically looked at the effects of cortisol, the intergenerational effects of stress may be transmitted via multiple mechanisms.

It is unclear whether the effects of cortisol exposure observed in this study have an adaptive value, or if they represent a non-adaptive physiological consequence of maternal stress. Reductions in swimming performance of fish have been linked to reduced migration success (Farrell *et al.*, 2008), increased susceptibility to predators (Walker *et al.*, 2005) and a general reduction in fitness, though few studies have directly tested this last relationship (Langerhans and Reznick, 2010). Additional work that focuses on the long-term ecological effects of embryonic cortisol exposure needs to be conducted in order to fully understand any fitness consequences on semelparous species.

This study has demonstrated that cortisol exposure during embryonic development can result in reduced aerobic swimming performance after hatch in Pacific salmon. Though the long-term fitness consequences of this result need to be explored, research has generally associated higher swimming performance with increased success of fitness-related activities, such as predator avoidance and migration. Traditionally, the management and conservation of migratory fish populations has relied primarily on ensuring sufficient fish return to the spawning grounds. However, if deleterious effects of maternal stress persist into the next generation, then conservation programs may benefit by taking the sub-lethal effects of stress on maternal condition into account during the development of management plans. These sub-lethal effects may have increasing importance in the future, given the projected influence of climate change and other anthropogenic stressors moving forward.

## Supplementary material

Supplementary material is available at *Conservation Physiology* online.

## Supplementary Material

Cortisol_Respiromentry_Supporting_Material_9-6-19_coz095

## Data accessibility

Data will be uploaded to the DryAd Digital Repository.
