# Erratum to: Simulated maternal stress reduces offspring aerobic swimming performance in Pacific salmon

**DOI:** 10.1093/conphys/coaa004

**Published:** 2020-02-05

**Authors:** Amanda I Banet, Stephen J Healy, Erika J Eliason, Edward A Roualdes, David A Patterson, Scott G Hinch

**Affiliations:** 1 Department of Biological Sciences, California State University, Chico, CA 95929, USA; 2 Pacific Salmon Ecology and Conservation Laboratory, Department of Forest and Conservation Sciences, Faculty of Forestry, University of British Columbia, Vancouver, BC V6T 1Z4, Canada; 3 Department of Ecology, Evolution, and Marine Biology, University of California, Santa Barbara, CA 93106, USA; 4 Department of Math and Statistics, California State University, Chico, CA 95929, USA; 5 Fisheries and Oceans Canada, Science Branch, Pacific Region, Co-operative Resource Management Institute, School of Resource Environmental Management, Simon Fraser University, Burnaby, BC V5A 1S6, Canada

*Conserv Physiol* 7(1): coz095; doi: 10.1093/conphys/coz095

An earlier version of this manuscript, which had not been approved by the author, was inadvertently published and therefore contained several minor formatting errors. The final version of this manuscript has now replaced this earlier version, and so these errors are no longer present. The Publisher apologises for this mistake, and any confusion caused.

