# Supplementary material for: Simulated maternal stress reduces offspring aerobic swimming performance in Pacific salmon
Source: Conserv Physiol. 2019 Dec 18;7(1):coz095. doi: 10.1093/conphys/coz095 (PMC6919300; doi:10.1093/conphys/coz095)
Supplement: Cortisol_Respiromentry_Supporting_Material_9-6-19_coz095 [file cortisol_respiromentry_supporting_material_9-6-19_coz095.docx]

**Simulated maternal stress reduces offspring aerobic swimming performance in Pacific salmon**

**SUPPORTING INFORMATION: EGG CORTISOL ASSAYS**

**Methods**

We used enzyme immunoassays (cortisol ELISA Kit; Neogen, Lexington, USA) to confirm that the cortisol bath treatments increased egg cortisol levels, and that these levels were within an ecologically relevant range. Assays were conducted on pink salmon and sockeye salmon eggs following methods similar to Sopinka et al. (2014). Approximately three hours after fertilization, three fertilized eggs were collected per treatment group from four pink salmon females and five sockeye salmon females. Eggs were homogenized in 1,200 μL of assay buffer using a dremel and 3mL of diethyl ether was added to the homogenate. We then vortexed the sample, let it sit for 30 minutes, and centrifuged it at 10,000g before moving it to a -80°C freezer for 30 minutes. The liquid phase was poured off and the ether was evaporated with N_2_. The remaining residue was reconstituted with 1,200 μL of assay buffer, heated in a heat block for 5 minutes at 65 °C, vortexed, heated for an additional 5 minutes, and vortexed again. 250 μL aliquots were then pipetted into Eppendorf tubes and stored in a -80°C freezer for later analysis. Samples were run in replicate following the directions provided by Neogen. Interassay coefficents of variation were 4.6% for pink salmon and 5.4% for sockeye salmon.

A linear mixed model was used to test for differences in treatment groups. Sibling pairs were treated as random variables to account for correlations between observations due to relatedness. P-values for mixed models were estimated via approximate F-tests with Kenward-Roger approximated degrees of freedom (Kenward and Roger, 1997). Species were analysed separately because the homogeneity of variance assumption was not met when analysed together.

**Results**

Egg cortisol was significantly higher in eggs that had been exposed to the cortisol baths (Figure S1: pink salmon: F_1,3_=14.43 p=0.03; sockeye salmon: F_1,4_=49.58, p=0.002).

**
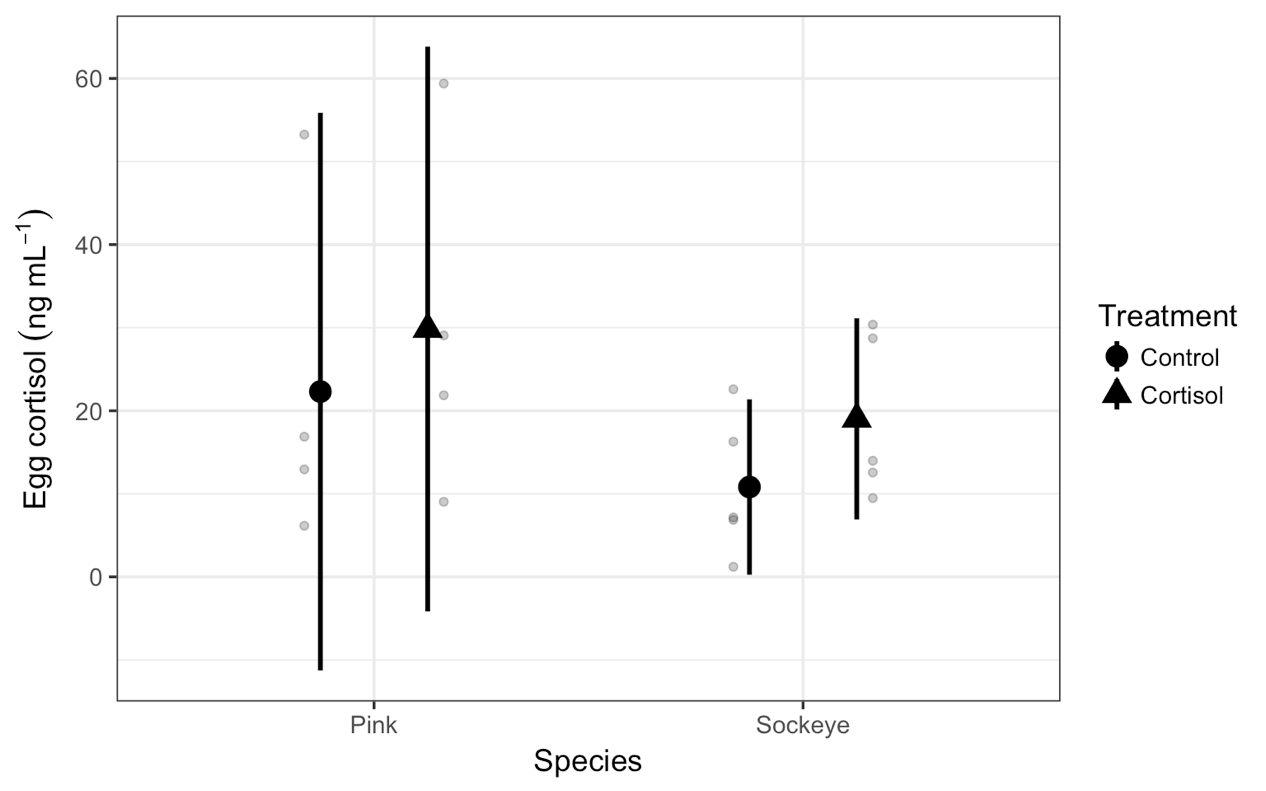
**

**Figure S1.** Egg cortisol levels from control and cortisol treatment groups in pink and sockeye salmon. Population means and 95% confidence intervals are shown in black. Gray circles represent individual fish. Cortisol treatment significantly increased egg cortisol levels in both species.

**Discussion**

Egg cortisol levels were significantly higher in eggs that were exposed to cortisol baths at fertilization. Mean egg cortisol levels from the treatment group were similar to egg cortisol levels observed from stressed Pacific salmon females in other studies (Stratholt *et al.*, 1997), increasing confidence that our treatments are ecologically relevant.

**References**

Kenward MG, Roger JH (1997) Small Sample Inference for Fixed Effects from Restricted Maximum Likelihood. *Biometrics* 53: 983–997.

Sopinka NM, Hinch SG, Middleton CT, Hills JA, Patterson DA (2014) Mother knows best, even when stressed? Effects of maternal exposure to a stressor on offspring performance at different life stages in a wild semelparous fish. *Oecol* 175: 493–500.

Stratholt ML, Donaldson EM, Liley NR (1997) Stress induced elevation of plasma cortisol in adult female coho salmon (Oncorhynchus kisutch), is reflected in egg cortisol content, but does not appear to affect early development. *Aquaculture* 158: 141–153.

**SUPPORTING INFORMATION: POST-HOC TABLES**

**Chinook** **Pink** **Sockeye**

**MO_2rest_ (mg O_2_ min^-1^ kg^-1^)**

Control 3.70 (3.10-4.31) 9 4.36 (3.75-4.96) 9 5.08 (4.56-5.60) 12

Cortisol 3.62 (3.01-4.22) 9 4.48 (3.87-5.08) 9 5.14 (4.62-5.67) 12

**MO_2max_** **(mg O_2_ min^-1^ kg^-1^)**

Control 10.62 (9.44-11.80) 9 17.50 (16.32-18.68) 9 12.39 (11.37-13.41) 12

Cortisol 8.32 (7.14-9.50) 9 15.16 (13.98-16.34) 9 11.60 (10.58-12.63) 12

**Aerobic Scope (mg O_2_ min^-1^ kg^-1^)**

Control 6.92 (5.59-8.24) 9 13.15 (11.82-14.47) 9 7.31 (6.16-8.46) 12

Cortisol 4.71 (3.39-6.03) 9 10.68 (9.36-12.00) 9 6.46 (5.32-7.61) 12

**Hematocrit (%)**

Control 37.9 (35.9-40.0) 17 48.2 (46.1-50.3) 19 40.4 (38.3-42.5) 14

Cortisol 38.0 (35.9-40.0) 18 46.6 (44.5-48.7) 17 40.7 (38.6-42.8) 14

**Hemoglobin (g L^-1^)**

Control 72.5 (69.2-75.8) 18 91.1 (87.8-94.5) 18 74.5 (70.9-78.1) 14

Cortisol 70.6 (67.3-74.0) 18 86.8 (83.5-90.1) 17 74.7 (71.0-78.3) 14

**Heart Mass (g)**

Control 0.0042 (0.0037-0.0047) 18 0.0060 (0.0055-0.0065) 24 0.0039 (0.0033-0.0044) 24

Cortisol 0.0041 (0.0035-0.0046) 18 0.0063 (0.0057-0.0068) 24 0.0036 (0.0030-0.0041) 24

**CS Activity (µmol min^-1^ g^-1^)**

Control 3.97 (3.45-4.48) 16 5.90 (5.47-6.34) 24 3.78 (3.34-4.22) 23

Cortisol 4.12 (3.60-4.63) 17 5.39 (4.96-5.83) 24 3.87 (3.44-4.31) 24

**LDH Activity (µmol min^-1^ g^-1^)**

Control 62.28 (46.45-78.11) 18 123.37 (109.66-137.08) 24 145.31 (131.60-159.02) 24

Cortisol 64.92 (49.10-80.75) 18 113.14 (99.43-126.85) 24 152.94 (139.23-166.65) 24

**Table S1.** Estimated marginal means, 95% confidence interval, and sample size for each species and treatment group. Data is listed in the following format: EMM (95% CI) n. EMMs for aerobic analyses are averaged over block (respirometer).

**Chinook-Pink** **Chinook-Sockeye** **Pink-Sockeye**

**MO_2rest_**

Control t_46.758_=-1.533, p=0.645 **t_46.668_=-3.467, p=0.014** t_46.833_=-1.823, p=0.462

Cortisol t_46.758_=-2.025, p=0.344 **t_46.668_=-3.844, p=0.005** t_46.833_=-1.674, p=0.555

**MO_2max_**

Control **t_43.452_=-8.327, p<0.001** t_43.363_=-2.293, p=0.219 **t_43.527_=6.605, p<0.001**

Cortisol **t_43.452_=-8.273, p<0.001** **t_43.363_=-4.250, p=0.001** **t_43.527_=4.594, p=0.001**

**Aerobic Scope**

Control **t_40.926_=-6.726, p<0.001** t_40.844_=-0.456, p=0.997 **t_40.995_=6.730, p<0.001**

Cortisol **t_40.926_=-6.449, p<0.001** t_40.844_=-2.028, p=0.345 **t_40.955_=4.865, p<0.001**

**Hematocrit**

Control **t_47.294_=-6.953, p<0.001** t_63.105_=-1.699, p=0.537 **t_63.734_=5.127, p<0.001**

Cortisol **t_45.893_=-5.982, p<0.001** t_65.511_=-1.788, p=0.480 **t_65.456_=3.940, p=0.003**

**Hemoglobin**

Control **t_57.122_=-7.793, p<0.001** t_76.312_=-0.826, p=0.962 **t_77.196_=6.511, p<0.001**

Cortisol **t_55.961_=-6.930, p<0.001**  t_77.267_=-1.573, p=0.618 **t_78.474_=4.862, p<0.001**

**Heart Mass**

Control **t_57.405_=-5.012, p<0.001** t_77.470_=0.815, p=0.964 **t_77.811_=5.452, p<0.001**

Cortisol **t_56.945_=-6.023, p<0.001**  t_77.906_=1.073, p=0.891 **t_79.282_=6.731, p<0.001**

**CS Activity**

Control **t_28.464_=-5.848, p<0.001** t_28.830_=0.579, p=0.992 **t_27.512_=7.009, p<0.001**

Cortisol **t_27.444_=-3.899, p=0.007** t_27.444_=0.735, p=0.976 **t_27.089_=5.029, p<0.001**

**LDH Activity**

Control **t_23.717_=-6.025, p<0.001 t_23.717_=-8.189, p<0.001**  t_23.717_=-2.337, p=0.219

Cortisol **t_23.717_=-4.755, p=0.001 t_23.717_=-8.680, p<0.001 t_23.717_=-4.239, p=0.004**

**Table S2.** Tukey adjusted p-values for hypothesis tests of estimated marginal means between species within each treatment group. Bold text indicates statistical significance. Sample sizes are available in table S1.
